# Efficacy of stem cell allograft in maxillary sinus bone regeneration: a randomized controlled clinical and blinded histomorphometric study

**DOI:** 10.1186/s40729-020-00222-w

**Published:** 2020-06-29

**Authors:** Josh Whitt, Mohanad Al-Sabbagh, Dolphus Dawson, Ehab Shehata, Moly Housley-Smith, Alejandro Tezanos, Ahmad Kutkut

**Affiliations:** 1 University of Kentucky College of Dentistry, Lexington, KY USA; 2 Division of Periodontology, University of Kentucky College of Dentistry, Lexington, KY USA; 3 Division of Oral and Maxillofacial Surgery, University of Kentucky College of Dentistry, Lexington, KY USA; 4 Department of Statistics, University of Kentucky College of Dentistry, Lexington, KY USA; 5 Division of Prosthodontics, University of Kentucky College of Dentistry, 800 Rose St. D646, Lexington, KY 40536 USA

**Keywords:** Maxillary sinus floor augmentation, Allograft, Stem cells, Sinus width, Dental implant

## Abstract

**Purpose:**

This study aimed to evaluate the quality and quantity of newly generated bone in the maxillary sinus grafted with stem cell-based allograft material.

**Methods:**

This study was a single site, prospective, blinded, randomized, and controlled clinical trial. Eleven subjects with 18 edentulous posterior maxillary sites requiring sinus augmentation for delayed implant placement using a lateral window approach were enrolled. At the time of sinus augmentation, test sinus was grafted with stem cell-based allograft (Osteocel Plus; NuVasive Therapeutics), while the control sinus was grafted with conventional cortico-cancellous allograft (alloOss; ACE Surgical). Cone beam computer tomography (CBCT) scan was taken before and 14 weeks post-sinus augmentation procedure, i.e., 2 weeks before implant placement. Thirty-six trephined core bone biopsies were harvested from the anterior and posterior grafted lateral-window osteotomy sites at the time of implant placement.

**Results:**

The results showed a statistically significant difference in the vital bone percentage between the test and the control groups at the posterior grafted sites (*p* = 0.03). There was no significant difference in the percentage of vital bone between the anterior and posterior grafted sites within the test and control groups (*p* > .05). The CBCT analysis showed that the maxillary sinuses at the posterior grafted sites were statistically wider than those at the anterior grafted sites in both groups (*p* < .05).

**Conclusions:**

Different allograft bone materials can be used in the maxillary sinus augmentation procedures. Stem cell allograft has more osteogenic potential with a better outcome in the wide posterior sinus.

## Introduction

The maxillary sinus is pyramidal in shape, where the anterior part is narrower than the posterior one. Antral dimensions are averaged 36–45 mm in height, 25–35 mm in anterior-posterior length, and 38–45 mm in latero-medial depth, with an average volume of 15 ml [1, 2].

Hard and soft tissue defects arise as a sequela of tooth extraction if no ridge preservation is attempted and maxillary sinus hyper-pneumatization poses a clinical challenge for proper implant placement [3]. Studies reported bone resorption of 0.7–1.5 mm and 4.0–4.5 mm at vertical and horizontal alveolar bone dimensions, respectively [4–6]. Most of these changes occurred during the first 3 months of healing [5]. Posterior maxillary tooth extraction causes inferior expansion (pneumatization) of the sinus in relation to other fixed landmarks. A radiographic study showed the enlarge of the sinus in an inferior direction by 1.83 ± 2.46 mm following tooth extraction. A more significant amount of expansion was measured after second molars extraction or extractions of two or more adjacent posterior teeth as compared to first molars [3]. To overcome such clinical limitations, several regenerative surgical techniques and materials have been introduced in the literature [3–6]. Fresh autogenous bone, which has high osteogenic potential, is considered the gold standard for bone regeneration in the maxillary sinus [7]. The donor site morbidities and low volume of available bone are the limitations of widespread use. Allograft materials have been used successfully as scaffolding materials in clinical studies; however, they lack cells with osteogenic potential [8].

Tissue engineering has been investigated in the augmentation of the vertically deficient maxillary ridge [9]. The standard tissue engineering entails in vitro culturing of an autologous cell onto a biological carrier (as a scaffold) before in situ implantation. Several factors can limit the in vitro cultures, such as the engineered tissue shape and size, cell integration, cell population, and the mechanical forces [10, 11]. A stem cell is an undifferentiated cell found among differentiated cells in a tissue or organ. It can renew itself and differentiate to a specific cell phenotype if exposed to the proper stimuli. Growth factors that are secreted from undifferentiated cells and have osteoinductive properties can be directly used to repair bone defects, which eliminate the limitations associated with cell culture [12]. The typical growth factors used for bone regeneration are bone morphogenic proteins (BMPs), fibroblast growth factors (FGFs), insulin-like growth factors I and II (IGF I/II), and platelet-derived growth factor (PDGF). These factors lead to different cellular responses, such as promoting cell adhesion, proliferation, migration, and osteogenic differentiation [7, 8, 13, 14]. A combination of growth factors and in vitro-expanded stem cells have been employed to enhance osteogenesis. This combination showed that stem cells could migrate effectively into and through growth factors and increase without deforming its cellular structure [7, 11–13].

Recent systematic reviews of human studies showed higher bone regeneration when applying mesenchymal stem cells compared with those with non-stem cell-based controls [15–18]. Multiple case series using allograft cellular bone matrix reported a high percentage of vital bone content after a relatively shorter healing time that encourages an earlier implant placement [19, 20].

Stem cell allograft has recently been available for the use in the osseous tissue generation [21]. The stem cell allograft, Osteocel Plus, NuVasive Therapeutics, has the scaffold and the native undifferentiated mesenchymal stem cells. The objective of the current study was to evaluate the quality and quantity of newly generated bone in the maxillary sinus grafted with stem cell-based allograft material. Also, the histomorphometric analysis was performed to compare the vital bone percentages in maxillary sinuses grafted with Osteocel Plus (NuVasive/Osiris Therapeutics, SAN DIEGO, CA) or alloOss (ACE Surgical, Brockton, MA).

## Methods

This study was a single-center, prospective, randomized, controlled clinical, and blinded histomorphometric study. The clinical trial was approved by the Medical Institutional Review Board at the University of Kentucky, Lexington, KY.

### Randomization

Eligible patients were randomly assigned to one of two treatment groups using a randomization table generated by a computer. The test group (nine sinuses) received sinus augmentation with Osteocel Plus, a stem cell allograft with a scaffold, and the control group (nine sinuses) underwent sinus augmentation with alloOss, a cortico-cancellous particulate allograft. Patients with bilateral sites in need of sinus augmentation had both the test and the control groups randomized as to which sinus received each group. Patients with single sites were randomized as to which group was administered to that site.

### Duration of study

This was a 2-year study. Follow-up examinations were at 14 days for suture removal, 14 weeks for follow-up CBCT, and 16 weeks for implant placement and core sample retrieval for histomorphometric analysis.

### Patient selection

Seven patients with bilateral edentulous posterior maxillary sites and four patients with unilateral edentulous posterior maxillary sites requiring sinus augmentation followed by implant placement were recruited from the University of Kentucky College of Dentistry and enrolled into the study by the study investigators.

## Study treatment

### Screening examination (visit 1)

Nine sinuses for each group were recruited from the University of Kentucky College of Dentistry by the study investigators and were screened for the study. Study participants were asked to read and sign the informed consent as well as encouraged to ask any questions that pertained to the study. Each patient received a copy of their informed consent once signed. Medical history and dental assessments were completed to rule out any systemic or local factors that would compromise the outcome of the study. Intra-oral and extra-oral soft tissue exams were performed at the screening visit along with a preoperative CBCT to plan the location of the lateral window sinus augmentation procedure. This was considered the standard of care before any sinus augmentation therapy.

To be included in this study, all subjects must understand the research and its requirements as well as be willing to participate, as shown by the voluntary signing of the written informed consent. The minimum age was at least 22-years old. Subjects also had to have at least one posterior site requiring grafting along with an implant restoration to replace one tooth in the edentulous site. Subjects had to be in satisfactory general health with no clinically significant positives on medical history as well as be available for all follow-up examinations.

Subjects were excluded from the study if they couldn’t undergo any oral surgery procedures.

Subjects were excluded if they smoked, had active periodontal disease, dental infections of the bone, uncontrolled diabetes, if they were undergoing chemotherapy or radiation, any autoimmune diseases, kidney or liver disease, or if they were pregnant or breastfeeding.

### Baseline surgical (visit 2) (Fig. 1)

Patients who qualified for the study and completed the informed consent were scheduled for the sinus augmentation procedure. All patients were offered to be sedated via moderate parenteral sedation using Midazolam and Fentanyl for the sinus augmentation procedure. After administration of local anesthesia (2% Lidocaine with 1:100,000 Epinephrine), a cresto-palatal incision was made using a 15C surgical blade (Integra LifeSciences Corporation), at the posterior maxillary site requiring augmentation. A full-thickness mucoperiosteal flap is reflected with two vertical releasing incisions. The lateral window osteotomy is created on the lateral wall of the maxillary sinus using a #8 round diamond bur (SS White), and Piezosurgery unit (Mectron Dental). The lateral window was opened to reflect the Schneiderian membrane ultimately (Fig. 2).

**Fig. 1:**
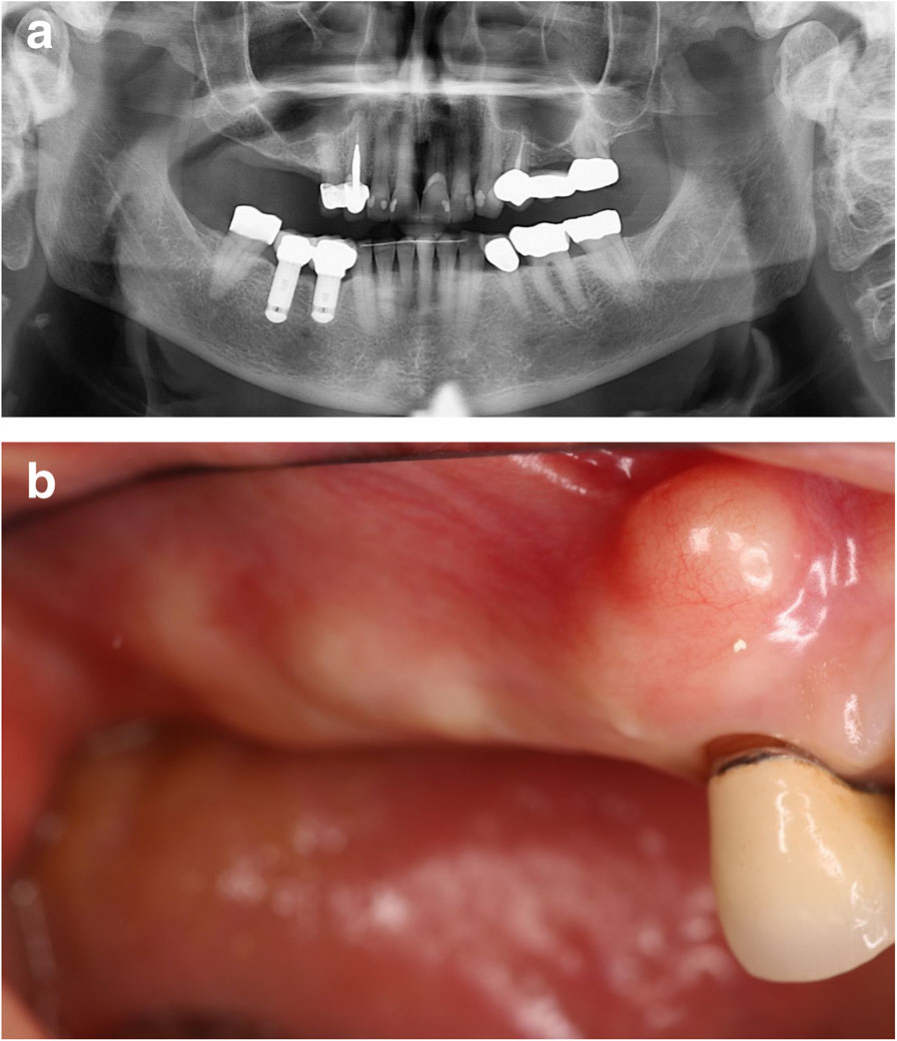
**a** Pre-operative panoramic X-ray. **b** Pre-operative edentulous posterior maxilla

**Fig. 2:**
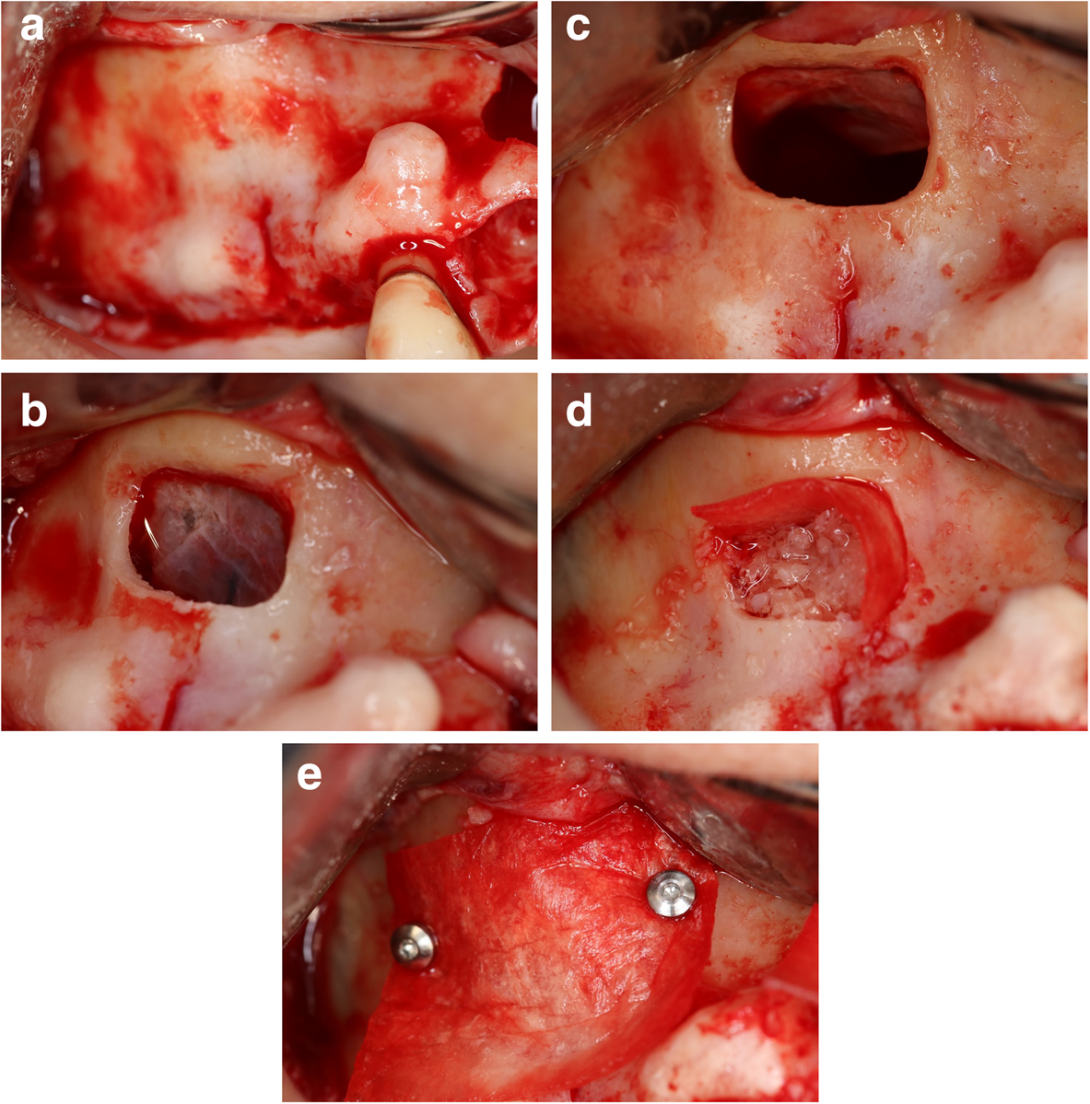
**a** Full-thickness flap reflected to access the lateral sinus wall. **b** Lateral window created. **c** Schneiderian membrane completely elevated to medial expose the medial wall. **d** Test sinus filled with Osteocel Plus allograft material. **e** Collagen resorbable membrane covers the window and stabilized with 2 tacks one anteriorly and the other one posteriorly

Once the Schneiderian membrane is reflected in the medial wall of the sinus, 2 g of Osteocel Plus was applied to the sinus of the test group, and 2 g of alloOss was used to the control sinus, densely packing after each application of the material. After each sinus is densely packed with graft material, the lateral window is covered with a resorbable collagen membrane (conFORM resorbable collagen membrane, ACE Surgical, Brockton, MA) to perform guided bone regeneration and prevent the ingrowth of soft tissue into the grafted site. Each membrane was then fixed with two tacks: one anterior and one posterior to the lateral window. All flaps were secured using interrupted sutures (3-0 chromic gut, Hu-Friedy Manufacturing, Co., LLC, Chicago, IL) with tension-free primary closure. Postoperative instructions were given to each patient and their escort, if sedated, after the surgical procedure.

All but one patient was prescribed augmentin 875/125 mg, one tablet every 12 h for 10 days, and was started 1 day before the surgery. The one patient allergic to Amoxicillin was prescribed Zithromax 250 mg (5-day PAK). They were taking two tablets on day one, then one tablet daily for days 2 through 5. Ibuprofen 600 mg was prescribed for postoperative analgesia. A total of 0.12% chlorhexidine gluconate was prescribed for patients to clean the surgical area gently three times daily for 2 weeks.

### Suture removal (visit 3) (Fig. 3)

Patients returned 2 weeks (14 days) after the sinus augmentation procedure. At this visit, the sutures were removed and wound healing was evaluated. Any adverse events were documented.

**Fig. 3:**
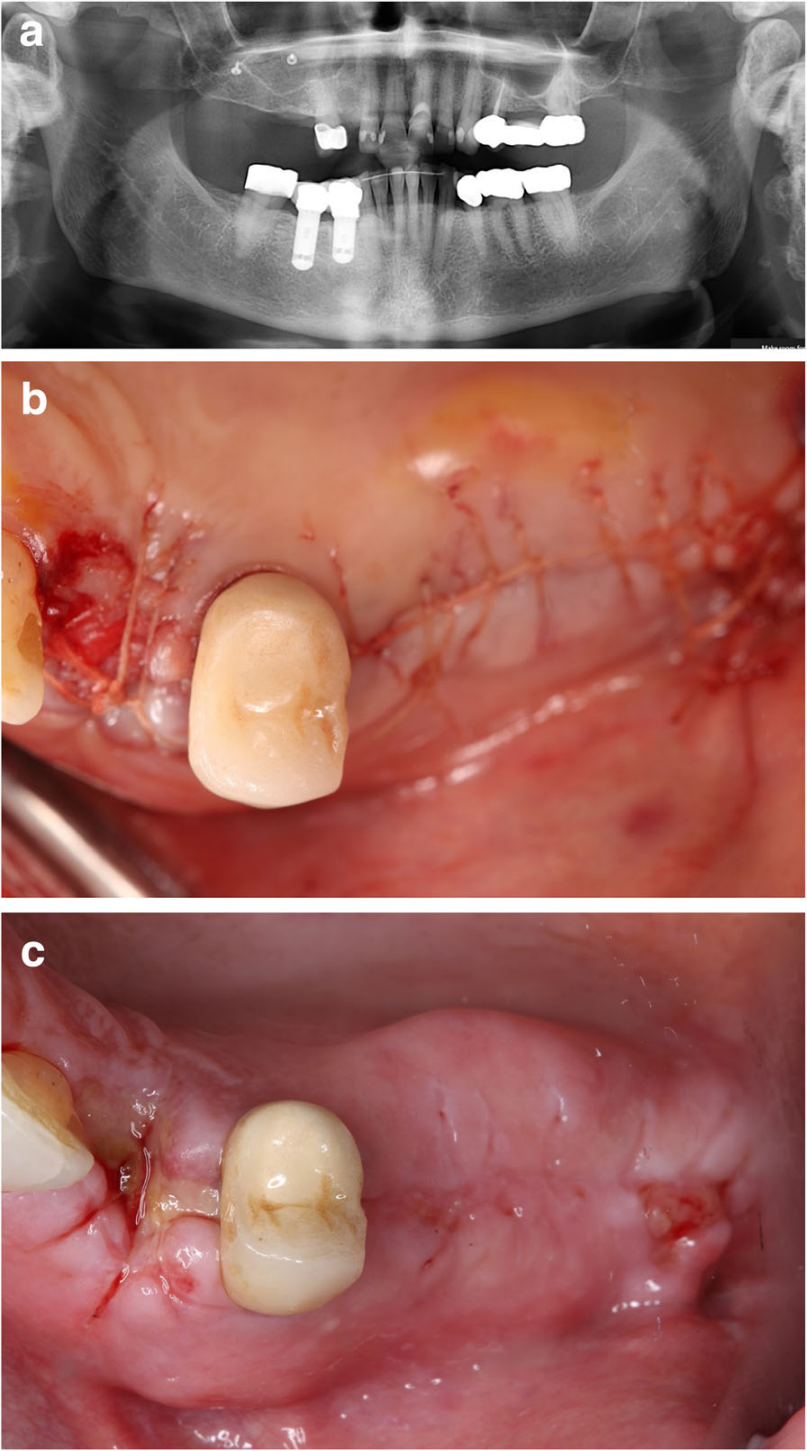
**a** Post-operative panoramic X-ray. **b** Tension-free primary closure. **c** Suture removal in 2 weeks follow-up visit

### Second CBCT (visit 4) (Fig. 4)

Patients were then allowed to heal for an additional 3 months (12 weeks after suture removal visit). At that time, a second CBCT was taken to allow for the planning of the implant placements.

**Fig. 4:**
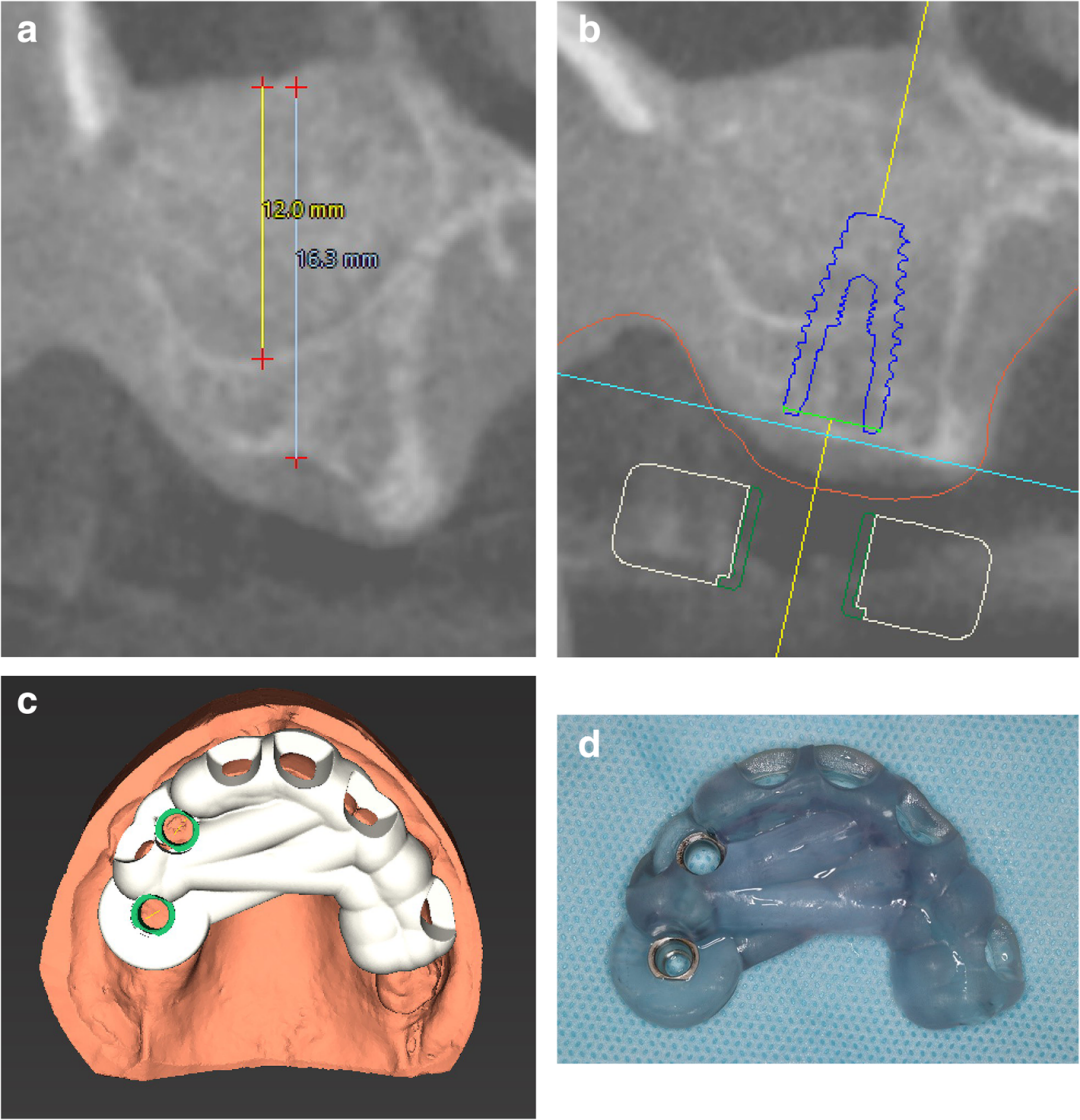
**a** 14 weeks follow-up CBCT to plan for implant placement. **b** Planning implant position into the grafted sinus using coDiagnosticX software. **c** Digital design for the surgical guide. **d** 3D-printed surgical guide

### Core samples and implant placement (visit 5) (Figs. 5 and 6)

Patients then returned 2 weeks after the second CBCT for the placement of the planned implants and the harvesting of the core samples from the lateral window site. After local anesthesia administration, a cresto-palatal incision was made, and a full-thickness flap reflected. Core samples of 2-mm diameter were harvested with a trephine bur at a speed of 1000 rpm and thorough irrigation. Samples were collected from the anterior and posterior sites of the lateral window where the bone healing was considered least mature in a horizontal direction (lateral to medial). Tacks placed to stabilize the resorbable membrane during the sinus augmentation procedure were used as a reference when harvesting the core samples. After core samples were collected, the tacks were removed. The harvested core samples were of such size not to compromise the ability to place the planned implants the same day. Core samples were placed in labeled formalin specimen tubes and taken to Oral Pathology. The osteotomies created by the trephine were grafted using alloOss bone graft.

**Fig. 5:**
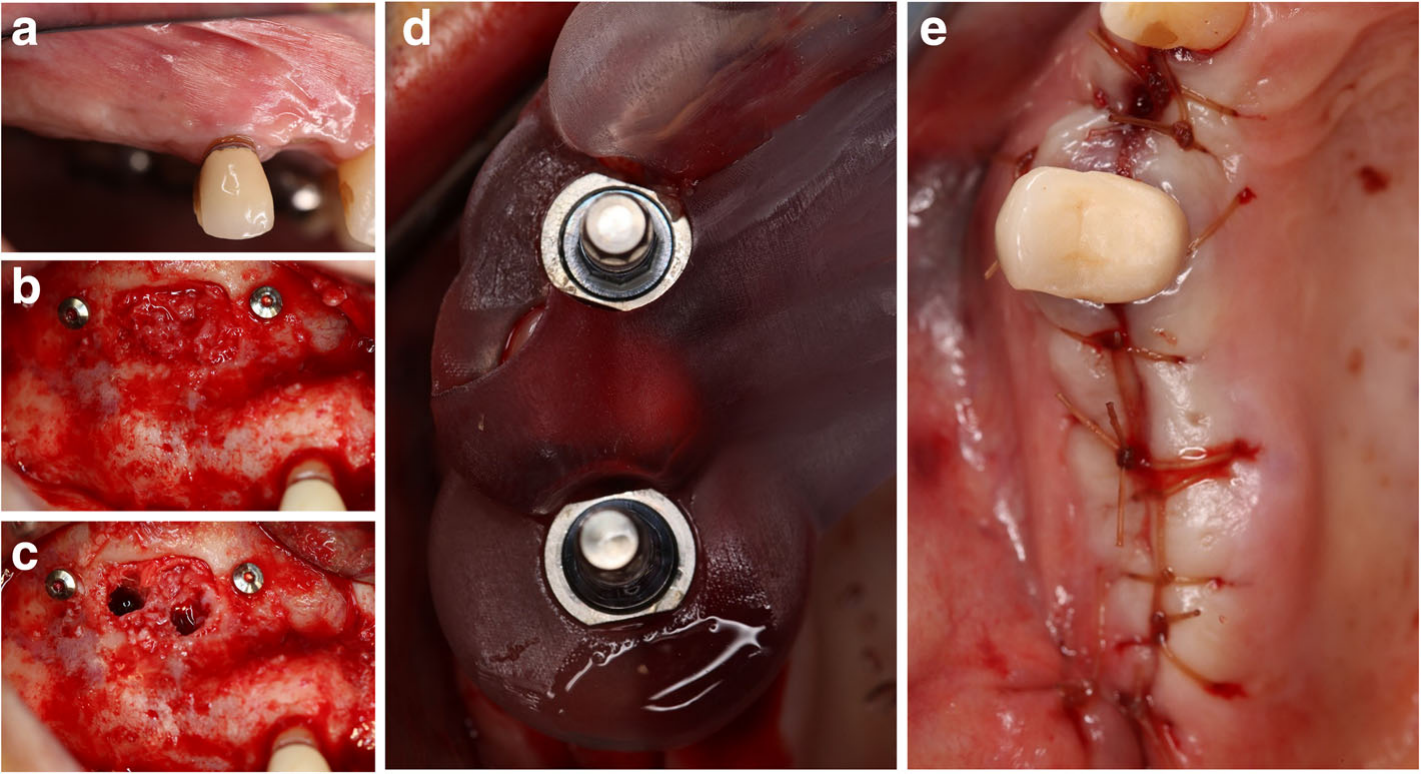
**a** 4 months of healing before implant placement surgery. **b** Full-thickness flap reflection to retrieve the core samples and tacks before implant placement. **c** Core samples retrieved from anterior and posterior sinus regions. **d** Fully guided implant placements using the surgical guide. **e** Tension-free primary closure after implant surgery

**Fig. 6:**
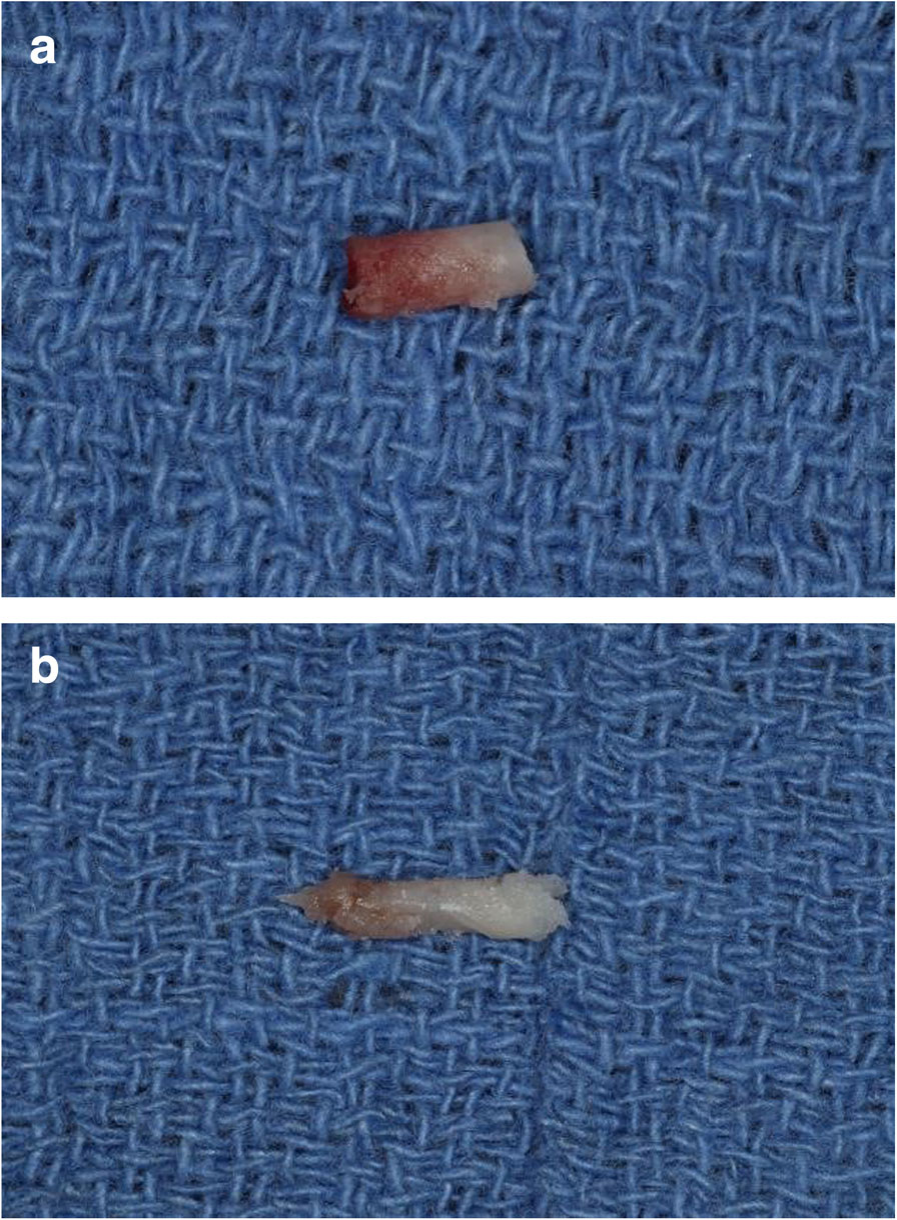
**a** and **b** Core samples retrieved from anterior and posterior sinus regions

Dental implants were placed through a digitally fabricated surgical guide with osteotomies created according to the implant manufacturer’s recommendations. All implants placed with good primary stability based on the torque value of at least 35 Ncm in grafted sinuses in both groups. Cover screws were placed, and flaps secured with 4-0 resorbable sutures. All patients were given postoperative care as wells as home care instructions. Patients were also advised to clean the surgical area gently with a Toothette Oral Swab moistened with 0.12% chlorhexidine gluconate four times daily for 2 weeks.

## Study measures

The treatment was considered successful if the bone core samples could be retrieved and enough bone was present for implants to be placed after 4 months of healing. Failure of treatment was considered when the bone core samples could not be harvested or implant placement could not be performed at 4 months of healing.

### Radiographic examination

A CBCT was taken as a baseline at the screening exam before the sinus augmentation procedure as a standard of care and at 3.5 months just before implant placement also as the standard of care. The height and width of the sinus were evaluated in the anterior and posterior sites of the planned lateral window. These two sagittal sections were used in the sinus measurement analysis. On each of the sagittal sections of the CBCT scan, two lines were drawn to measure the dimension of the sinus. One line measured the horizontal width, and the other line measured the vertical height. The horizontal width of the sinus was measured at a vertical height of 10 mm from the sinus floor based on the standard average length of implants used in implant dentistry.

### Histomorphometric analysis

Thirty-six trephined core samples were retrieved surgically from the grafted lateral window at the time of implant placement (visit 5). One core was retrieved from the anterior area of the sinus, 4 mm distal to the anterior tack, which was used for membrane fixation, and the other sample was retrieved from the posterior region, 4 mm mesial to the posterior tack which was used for membrane fixation.

Core samples were placed in special biopsy tubes containing 10% neutral buffered formalin for fixation at least 24 h before decalcification in Decal Stat™ acid (Decal Chemical Corporation, NY USA**)** for 1 h, dehydrated in a graded series of ethanol, and embedded in Ameraffin tissue embedding medium wax. Harvested core samples were sent to a research laboratory (University of Kentucky, Lexington, KY) for light microscopy histologic slides preparation. The specimens were cut in the apicocoronal plane to obtain three 6-μm-thick sections that were used for light microscopic examination and histomorphometric analysis.

### Photographic documentation

For each visit, photographs were taken using a digital camera for step-by-step documentation and monitoring of the healing process.

### Adverse events

Patients were evaluated within 2 weeks for suture removal and evaluation of wound healing and 14 weeks following the sinus augmentation procedure for implant placement and core sample retrieval. Patients were monitored for any adverse reactions, i.e., complications or failures throughout the study, and there were no adverse events recorded related to the study protocol.

## Results

All statistical analyses were completed in SAS 9.4 (SAS Institute Inc., Cary, NC, USA). Significance level set at 0.05.

The results show that the test group has significantly higher posterior vital bone than the control group (*p* value 0.03) (Table 1). In the control and test group, the anterior sinus width is significantly higher than the posterior sinus width (*p* value 0.0002). The differences between sinus width and gender were reported in Table 2.

**Table 1:** The difference between test and control group on four variables: anterior sinus width and vital bone, posterior sinus width, and vital bone

Test group (osteocell)				Control group (alloOss)			
Anterior sinus width	%Vital bone	Posterior sinus width	%Vital Bone	Anterior sinus width	%Vital bone	Posterior sinus width	%Vital bone
**12.35 ± 1.44 mm**	**44.11% ± 0.33**	**17.27 ± 2.28 mm**	**50.12% ± 0.35**	**14.15 ± 1.54 mm**	**34.14% ± 0.35**	**18.49 ± 2.68 mm**	**24.45% ± 0.23**

**Table 2:** The difference in sinus width by gender

Gender	Mean difference	SD	Std Err mean	Lower 95%	Upper 95%
Female	−2.42	0.91	0.64	−10.55	5.71
Male	−0.686	1.30	0.58	−2.31	0.93

When comparing differences in sinus width by gender using a *t* test, there was no evidence of statistically significant difference (*p* value = 0.1435). When comparing differences in sinus width by age using linear regression, there was no evidence of statistically significant difference (*p* value = 0.7631). When comparing vital bone differences by age using linear regression, there was no evidence of statistically significant difference (*p* value = 0.1821). When comparing vital bone differences by age using a *t* test, there was evidence of statistically significant difference (*p* value = 0.0071). Females have a higher averaged vital bone difference from pre to post-op sinus augmentation.

Additional file 1: Diagram 1 shows no significant difference in the correlation between sinus width and gender (*p* value = 0.14). Additional file 2: Diagram 2 shows no significant difference in sinus width and age using linear regression (*p* value = 0.76). Additional file 3 Diagram 3 shows the significant difference between gender and vital bone. Females have higher averaged vital bone difference from pre- to post-op sinus augmentation (*p* value = 0.007). Additional file 4: Diagram 4 shows no significant difference when comparing vital bone and age using linear regression (*p* value = 0.18).

### Histologic findings

**Fig. 7:**
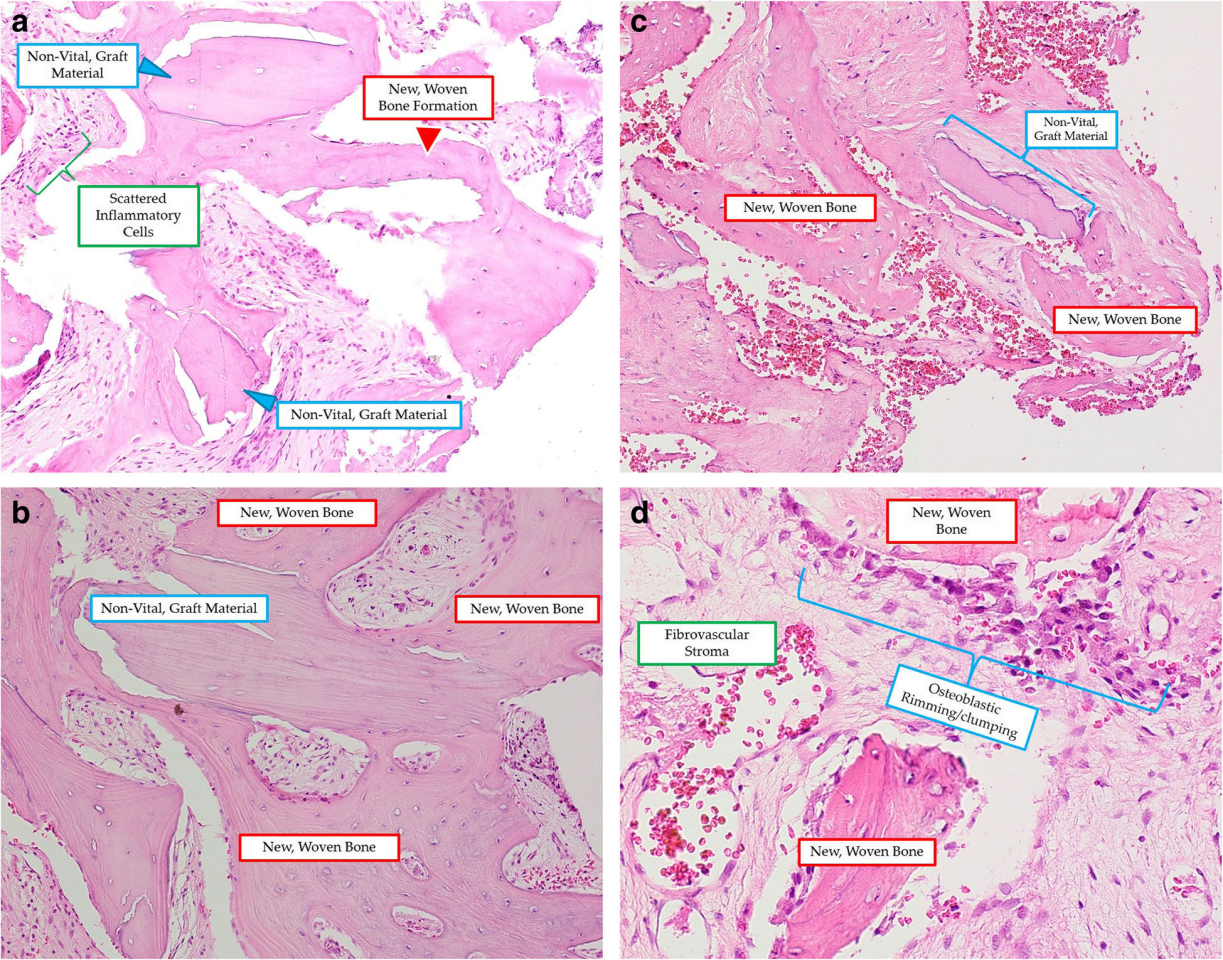
**a**-**d** Histomorphometric findings from each site and group

Figure 7 shows the histomorphometric results with vital bone in anterior and posterior sinus areas in both groups.

## Discussion

This investigation histomorphometrically compared alloOss (ACE Surgical) (control group), a cortico-cancellous allograft, to Osteocel Plus (NuVasive/Osiris Therapeutics), an allograft cellular bone matrix (test group) by comparing the percentage of vital bone present at 4 months of healing. The results showed that the test group (Osteocell) had a significantly higher vital bone in the posterior region than the control group (alloOss).

Tatum initially described maxillary sinus augmentation via the lateral window techniques in the mid-seventies [7]. The lateral window is still one of the most frequently used treatment modalities to increase the vertical alveolar bone height of the posterior maxilla before implant rehabilitation.

Kutkut et al. [22] presented a clinical recommendation when treatment planning maxillary sinus augmentation based on sinus width and blood supply. The report suggests that a classification technique of the maxillary sinus anatomy based on the mean sinus width of 15.2 mm. Sinus width < 15.2 mm is proposed as a narrow sinus cavity and classified as a quicker bone regeneration “QBR” in terms of bone healing after sinus augmentation procedure. On the other hand, sinus cavity wider than 15.2 mm is proposed as a wide sinus cavity and was classified as a slower bone regeneration “SBR” in terms of bone healing.

When the width increased, the distance of the blood supply also increased and made less blood supply available to the grafted site affecting vital bone formation. The reasons for the correlation between height and width may be related to the healing factors and angiogenesis that occurs after sinus augmentation surgery [22]. Because the blood supply to the sinus is critical for healing and bone formation, any factor that brings this supply closer to the graft material would be expected to improve healing [23–25]. For example, a sinus with a narrow horizontal width, closer proximity of surrounding walls, would present a better source of blood supply to the grafted bone replacement particles, which are osteoconductive. A smaller height to width ratio would allow cells and healing proteins less distance to migrate during the bone regeneration process, and this was evident in the control group in this study where the vital bone percentage was doubled in the narrow anterior sinus compare to wide posterior sinus after 4 months of healing [22, 26].

Regarding the blood supply to the sinus cavity, there are three primary arteries supplying blood to the maxillary sinus: the posterior superior alveolar artery, the infraorbital artery, and the posterior lateral nasal artery. These arteries are the branches of the maxillary artery. The posterior lateral nasal artery supplies the medial wall, which plays an essential role in vital bone formation. Therefore, the closer the medial wall is to the lateral wall (narrow sinus), the better the available blood supply to the graft material [27, 28]. Besides, this would increase the surface area of the surrounding bone from which new bone formation occurs. This is the reason why the Schneiderian membrane must be elevated entirely from the medial wall when performing a sinus lift procedure. Margolin et al. [29] evaluated the performance of graft materials at different heights. They were able to visualize on the CBCT scans that the mineralization was more rapid near the floor of the sinus and along the peripheral regions. This pattern of mineralization was anticipated based on the source of vascular ingrowth and bone-forming cells. This investigation presents a classification technique, which is consistent with these findings when the width and height of the sinus are considered.

In the test group of this study, the percentage of the vital bone of wide sinus morphology was higher than the narrow anterior sinus. This is because of the stem cells-based allograft Osteoinduction effect was more materials existed in the wide posterior sinus. The sinus anatomy is pyramidal in shape. The anterior part of the sinus is narrower than the posterior region. Consequently, the bone remodeling may be faster anteriorly, and therefore implant placement may be more predictable in the anterior maxilla than the posterior area.

Moreover, the success rate may be expected to be higher in narrow or (QBR) sinus morphology. This research is based on the distance of width and height of the maxillary sinus. The actual amount of blood supply will be determined by the exposed surface area of the medial wall of the maxillary sinus. This study confirmed the proposed classification [21] that correlates to the amount of vital bone and width of the sinus and provide evidence that the most critical factors inclusive of the type of the graft material that may cause differences in the amount of new vital bone formation in grafted sinuses is the anatomy of the maxillary sinus.

Recent systematic reviews have assessed implant survival in maxillary sinus augmentation with different grafting materials and found that the implant survival rate was the same regardless of the grafting material or the time of implant placement [15–17]. Also, the reviews have concluded that autogenous bone grafting has resulted in the highest amounts of newly formed bone. Various bone substitutes, such as xenografts, allografts, and alloplasts, are an excellent alternative to autogenous grafting [18]. Total bone volume after sinus augmentation revealed a significantly higher mineralized bone during the early healing phase with autogenous grafting compared to that of other grafting materials used alone or in combination with autogenous grafting [19]. However, when higher than 9 months of healing was achieved, there was no statistically significant difference between different grafting materials [18]. Conversely, it appears that the volume of the maxillary sinus does not influence the contraction or dimension of a particular bone graft placed; however, in general, there seems to be an overall decrease in graft dimensions over time [20, 21, 30]. The results of this study may be due then an increase in the osteogenic potential of the test bone graft material.

## Conclusion

Sinus augmentation in the posterior maxilla can be completed with many grafting materials as well as combinations of grafting materials. Sinuses augmented with Osteocel Plus (NuVasive/Osiris Therapeutics) had a statistically significant higher vital bone percentage after just 4 months of healing in the posterior aspect of the sinus compared to the posterior aspect of a sinus grafted with alloOss (ACE Surgical). More cases and monitoring of the future survival of implants placed in these augmented sinuses are needed to verify these results.

## Supplementary information

**Additional file 1.** Diagram1. Correlation between sinus width and gender.

**Additional file 2.** Diagram 2. Correlation between sinus width and age.

**Additional file 3.** Diagram 3. Correlation between vital bone and gender.

**Additional file 4.** Diagram 4.Correlation between vital bone and age.

## Data Availability

Yes, available.

## Abbreviations

CBCT: Cone beam computer tomography
BMPs: Bone morphogenic proteins
FGFs: Fibroblast growth factors
IGF I/II: Insulin-like growth factors I and II
PDGF: Platelet-derived growth factor
QBR: Quicker bone regeneration
SBR: Slower bone regeneration
