# Supplementary figures and images for: Efficacy of stem cell allograft in maxillary sinus bone regeneration: a randomized controlled clinical and blinded histomorphometric study
Source: Int J Implant Dent. 2020 Jun 29;6:25. doi: 10.1186/s40729-020-00222-w (PMC7321846; doi:10.1186/s40729-020-00222-w)

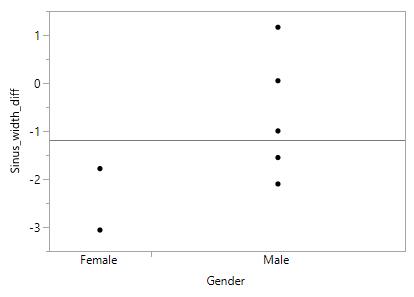

Supplement: Supplementary file 1 — Additional file 1. Diagram1. Correlation between sinus width and gender. [file 40729_2020_222_MOESM1_ESM.jpg]

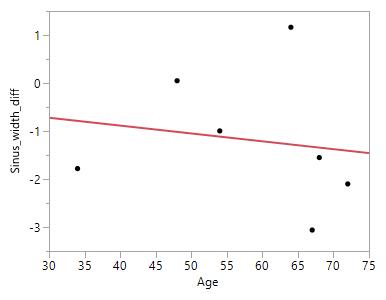

Supplement: Supplementary file 2 — Additional file 2. Diagram 2. Correlation between sinus width and age. [file 40729_2020_222_MOESM2_ESM.jpg]

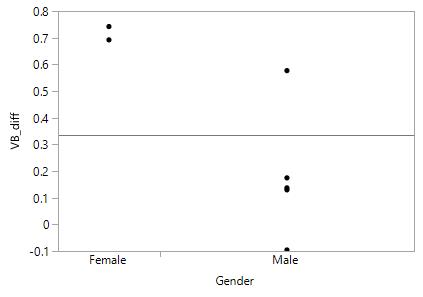

Supplement: Supplementary file 3 — Additional file 3. Diagram 3. Correlation between vital bone and gender. [file 40729_2020_222_MOESM3_ESM.jpg]

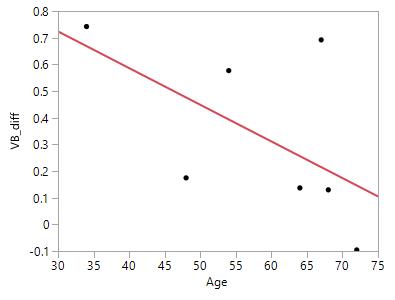

Supplement: Supplementary file 4 — Additional file 4. Diagram 4.Correlation between vital bone and age. [file 40729_2020_222_MOESM4_ESM.jpg]
